# Advancing beyond the system: telemedicine nurses’ clinical reasoning using a computerised decision support system for patients with COPD – an ethnographic study

**DOI:** 10.1186/s12911-017-0573-7

**Published:** 2017-12-28

**Authors:** Tina Lien Barken, Elin Thygesen, Ulrika Söderhamn

**Affiliations:** 1 Centre for eHealth, Centre for Care Research, Southern Norway, Department of Health and Nursing Sciences, Faculty of Health and Sport Sciences, University of Agder, Post box 422, 4604 Kristiansand, Norway; 2 Centre for Care Research, Southern Norway, Department of Health and Nursing Sciences, Faculty of Health and Sport Sciences, University of Agder, Post box 422, 4604 Kristiansand, Norway

**Keywords:** Computerised decision support system, Chronic obstructive pulmonary disease, Decision-making, Ethnography, Nursing, Reasoning, Telemedicine, Qualitative

## Abstract

**Background:**

Telemedicine is changing traditional nursing care, and entails nurses performing advanced and complex care within a new clinical environment, and monitoring patients at a distance. Telemedicine practice requires complex disease management, advocating that the nurses’ reasoning and decision-making processes are supported. Computerised decision support systems are being used increasingly to assist reasoning and decision-making in different situations. However, little research has focused on the clinical reasoning of nurses using a computerised decision support system in a telemedicine setting. Therefore, the objective of the study is to explore the process of telemedicine nurses’ clinical reasoning when using a computerised decision support system for the management of patients with chronic obstructive pulmonary disease. The factors influencing the reasoning and decision-making processes were investigated.

**Methods:**

In this ethnographic study, a combination of data collection methods, including participatory observations, the think-aloud technique, and a focus group interview was employed. Collected data were analysed using qualitative content analysis.

**Results:**

When telemedicine nurses used a computerised decision support system for the management of patients with complex, unstable chronic obstructive pulmonary disease, two categories emerged: “the process of telemedicine nurses’ reasoning to assess health change” and “the influence of the telemedicine setting on nurses’ reasoning and decision-making processes”. An overall theme, termed “advancing beyond the system”, represented the connection between the reasoning processes and the telemedicine work and setting, where being familiar with the patient functioned as a foundation for the nurses’ clinical reasoning process.

**Conclusion:**

In the telemedicine setting, when supported by a computerised decision support system, nurses’ reasoning was enabled by the continuous flow of digital clinical data, regular video-mediated contact and shared decision-making with the patient. These factors fostered an in-depth knowledge of the patients and acted as a foundation for the nurses’ reasoning process. Nurses’ reasoning frequently advanced beyond the computerised decision support system recommendations. Future studies are warranted to develop more accurate algorithms, increase system maturity, and improve the integration of the digital clinical information with clinical experiences, to support telemedicine nurses’ reasoning process.

## Background

Traditional nursing practice is incorporated in the future of healthcare technology [1, 2] to develop new roles responsive to changing needs [3, 4]. Telemedicine (TM), which can be defined as ‘information, communication, and monitoring technologies which allow healthcare providers to remotely evaluate health status, give educational intervention, or deliver health and social care to patients in their homes’ ([5] p. 2813), has demonstrated potential for advanced nursing practice regarding efficiency [6] and quality of care [7–9]. However, TM involves the provision of advanced nursing care in a new clinical environment [10], which enables remote care [10, 11] for patients with chronic diseases, such as chronic obstructive pulmonary disease (COPD) [12]. COPD is a serious and progressive chronic disease [12] representing a high symptom burden [13]. Chronic diseases often involve subtle [10] and unpredictable disease developments [9, 14] that require complex disease management strategies [2].

Complex clinical practice entails that nurses use appropriate clinical reasoning skills [15]. Nurses’ clinical reasoning can be defined as: “…the cognitive processes and strategies that nurses use to understand the significance of patient data, to identify and diagnose actual or potential patient problems, to make clinical decisions to assist in problem resolution, and to achieve positive patient outcome” ([16] p. 236). Hence, clinical reasoning is the sum of critical thinking and decision-making processes associated with clinical practice [17], where critical thinking is based on nurses’ careful, deliberate thoughts in different clinical settings [18]. Consequently, it is essential that within TM, clinical reasoning and decision-making are supported.

Computerised decision support systems (CDSS) use algorithms to produce patient-specific assessments [19] and decision-making recommendations to support reasoning [20, 21], helping nurses to make better clinical decisions [16] and improving patient care [19]. CDSS use has increased in recent years [21], but ambiguity regarding its adoption, implementation [21, 22] and accuracy [23] remains. Various factors, such as familiarity with the patient [24–27], the patient’s condition, and the CDSS technology used, can affect the decision-making process [10, 27]. Moreover, CDSS use for long-term management involves complex decision-making [21], which requires advanced training especially when used in a TM setting [1].

CDSSs have been used in hospitals [15, 28, 29], primary [27] and chronic care setting [30, 31], and for phone-based counselling [32–34]. However, few studies have evaluated TM nurses’ reasoning using these systems to manage patients with COPD in the primary care setting. Additionally, these systems are developed and implemented based on a limited understanding of clinical work and decision-making [22]. Therefore, the context in which CDSSs are used must be evaluated [32, 35], for example, for nurses’ reasoning [16, 36] and decision-making processes [37–39] during the provision of care. The present study combined different qualitative data collection methods consisting of participatory observations, the think-aloud technique, and a focus group interview, to gain a better understanding of TM nurses’ clinical reasoning using a CDSS when managing patients with COPD.

## Methods

### Aim

The purpose of the study was to explore the process of TM nurses’ clinical reasoning when using a CDSS for the management of patients with COPD. The factors influencing the clinical reasoning and decision-making processes were investigated.

### Design

An ethnographic approach [40] was chosen to investigate the reasoning and decision-making processes of TM nurses, in order to gain insight into their subjective world in their natural settings [41, 42], and to provide a nuanced picture of the field [42, 43]. Participatory observations [40], the think-aloud technique [44] and one focus group interview [45] was employed.

### Setting and participants

This study was conducted at a telemedicine centre (TMC) in a municipality in southern Norway, between October 2015 and February 2016. The TMC was established as part of a larger European project; ‘United4Health’ (U4H). The U4H project explored a TM intervention for patients with COPD, who had been hospitalised for exacerbation and discharged to their home [46, 47].

The present study focused on the TM nurses employed at the TMC, in order to monitor and assess COPD patients. The TMC employed seven registered nurses that managed COPD patients from several municipalities, and was open from 8 am to 3 pm, Monday to Saturday. The TMC was operated and managed by a single nurse at any given time, and could manage up to 12 patients daily. Registered nurses who were employed at the TMC were included in our study sample, and asked to participate. Three of seven nurses agreed to participate. The four nurses who did not wish to participate were employed part-time with weekend shifts once a month. All participants were female and aged between 27 and 46. Table 1 shows the participant characteristics.

**Table 1:** Participant characteristics

Participant	Nurse 1	Nurse 2	Nurse 3
Nursing experience	6 years	8 years	23 years
Telemedicine experience	2 years	1.5 years	4 months
Employed at the centre	80%	60%	45%
Continuing education	Master’s degree in health and social informatics	–	Geriatrics

#### Technical equipment and computerised decision support system

The technical equipment used in the intervention consisted of a pulse oximetry device for daily measuring of oxygen saturation and heart rate. In addition, a tablet application which included an electronic questionnaire to gather patients’ daily subjective symptoms was used. The questions concerned issues such as well-being, breathlessness, sputum characteristics and medication use (Table 2). Patient measurements and reported subjective symptoms were uploaded by the tablet application and transmitted securely and wirelessly to a data server at the TMC. TM nurses at the TMC monitored and assessed the transmitted data using a CDSS. In addition, TM nurses followed up patients using real-time video-conference supported by the tablet application.

**Table 2:** Daily questionnaire performed by COPD patients, and evaluated by TM nurses [48]

Question	Response
1 How do you feel today?	As usual Worse Much worse
2 How is your breathing today?	As usual Worse Much worse
3 How is your amount of sputum today?	As usual Worse Much worse
4 What is the colour of your sputum today?	No sputum/Clear/White/ Yellow/Green/Brown
5 Are you using rescue medication/nebuliser^a^ or oxygen today?	No As usual More than usual Much more than usual
6 Have you started up with additional antibiotics after last discharge?	No Yes
7 Have you started up with new Prednisolone^b^ after last discharge?	No Yes

^a^Rescue medication/nebuliser; inhalation medicine for breathing treatment

^b^Prednisolone; a steroid, used to treat various conditions including breathing disorders

The CDSS was developed by medical and technical experts employed at the local hospital and the university. It provided the TM nurses with a daily overview over patients’ health status and condition, and was used to support their reasoning and decision-making processes. The reported patient measurements and subjective symptoms (see Table 2) transmitted from the tablet application, were the basis for an automatic calculation with a specific algorithm that resulted in display of colour codes [47, 48]. The colour codes were either green, yellow, or red, indicating warning alerts on patients’ health status and disease development (see Table 3). The thresholds for clinical measurements, such as oxygen saturation and heart rate, were predefined for each patient based on their reference values at hospital discharge, and these were used for the following day-to-day monitoring. To increase the sensitivity of the system, a cut-off value for “yellow” status was added to indicate early warning indicator for health deterioration. The cut off values for “red” alert was developed and based on existing empirical clinical measurements and algorithms used in the UK [48]. If the clinical measurements fell out of the normal (i.e., individual) reference range, or if the patients answered “worse” for subjective symptoms (see Table 2), the colour code became yellow or red depending on severity of the health change (see Table 3). In addition, if health data was not provided on a given day, the nurse would contact the patient.

**Table 3:** Algorithm for CDSS [48]

Colour displayed at CDSS	Indication of health symptoms
Green	Stable patient: Self-reported health symptoms unchanged or improved. Oxygen saturation and heart rate within acceptable range, compared to individual reference values.
Yellow	Unstable patient indicating change that need follow-up: oxygen saturation and/or heart rate indicate deterioration from previous day or from hospital discharge. Yellow alert is triggered. Change indicating: increase in heart rate more than 10 beats/min, reduction in saturation of approximately 5%, an answer to question 1, 2, 3 or 4 defined as “worse”, or question 5 answered with “more than usual” (Table 2).
Red	Unstable patient indicating severe change or critical condition: oxygen saturation and/or heart rate or self-reported health symptoms (Table 2) indicating significant deterioration from previous day or from hospital discharge. Red alert is triggered. Change indicating: increase in heart rate more than 15 beats/min, reduction in saturation lower than 6%, or daily questionnaire (1–5) answered with “much more than usual” (Table 2).

*CDSS* computerised decision support system

### Data collection

Data were collected through using participatory observations [40] (major data source), the think-aloud technique [44], and a focus group interview [45]. The fieldwork enabled observations of the nurses when performing clinical reasoning, decision-making and video consultations with the patients. Approximately 60 patient consultations were observed during 60 fieldwork hours; Nurse 1 was followed for 30 h, and Nurses 2 and 3 were followed for 15 h each. The fieldwork was then completed according to the wishes of the participants. Observations were conducted between 8.30 and 11.30 am, on weekdays.

The think-aloud technique [44] facilitated insight into participant verbalisations, clinical information processing, and performance of different tasks. Each nurse expressed their thoughts aloud, explaining each step in their reasoning process. Probing questions such as “please, could you elaborate your reasoning” or “please, could you continue to think aloud” were asked to gather information and clarifications during each patient assessment.

Field notes consisting of observations, actual incidences, reflections, and interpretations were made during and after the day’s observations [41, 49]. Details of setting and human behaviour were recorded to provide a complete and representative record of the field investigated [40]. Approximately 134 A4 pages were written during the fieldwork.

After fieldwork completion, a focus group interview [45] was conducted to gain insight into the participants’ reasoning and decision-making processes experiences when using the CDSS. A semi-structured interview approach was utilised [50]. Open-ended questions focused on the use of the CDSS and the nurses’ reasoning and decision-making processes. Example questions included “What is your experience of the CDSS in supporting and improving the decision-making process when assessing patients?” and “Could you give examples of how you used your expertise to make decisions relating to the patient?”. A moderator was present during the one hour and thirty-minute discussion, and the collected data was transcribed verbatim.

### Data analysis

Data were analysed using qualitative manifest and latent content analysis [51], as follows: in the manifest analysis 1) the text was repeatedly read to gain understanding of the whole, 2) and was divided into meaning units, 3) meaning units were condensed to reflect participants’ statements and observational data, 4) condensed meaning units were abstracted and grouped into codes with common denominators to give an outline of the data, and 5) codes were organized into categories and subcategories. The latent analysis involved an interpretation of the underlying meaning of the text, leading to an overall theme [51]. The material was sorted using NVivo 10 [52]. An example of the analytical process is presented in Table 4.

**Table 4:** Example of the analytical process

Meaning unit	Condensed meaning unit	Code	Sub-category	Category
I cannot look at that alone [the CDSS recommendation] … What colour is it [the system alert]? I need to look at the patient’s background, comorbidities, and recent health status [disease development].	The nurse overrides the recommendation by gathering extended clinical data to assess health change	Gathering and searching for extended clinical data to assess the recommendation	Assessing the recommendation	The process of TM nurses’ reasoning to assess health change

*CDSS* computerised decision support system, *TM nurses* telemedicine nurses

## Results

Two categories emerged from the analysis: “the process of TM nurses’ reasoning to assess health change” and “the influence of the TM setting on nurses’ reasoning and decision-making processes”. The overall theme, “advancing beyond the system”, represented the connection between the nurses’ reasoning process and the TM setting and work (Table 5).

**Table 5:** Presentation of the overall theme, categories and sub-categories of the nurses’ reasoning and decision-making processes

Overall theme	Advancing beyond the system	
Categories	The process of TM nurses’ reasoning to assess health change	The influence of the TM setting on nurses’ reasoning and decision-making processes
Sub-categories	Assessing the CDSS recommendations	Environment
	Mapping, combining and interpreting data to reach a pre-decision	Technology
	From pre-decision to shared decision-making	

*CDSS* computerised decision support system, *TM* telemedicine

### The process of TM nurses’ reasoning to assess health change

**Fig. 1:**
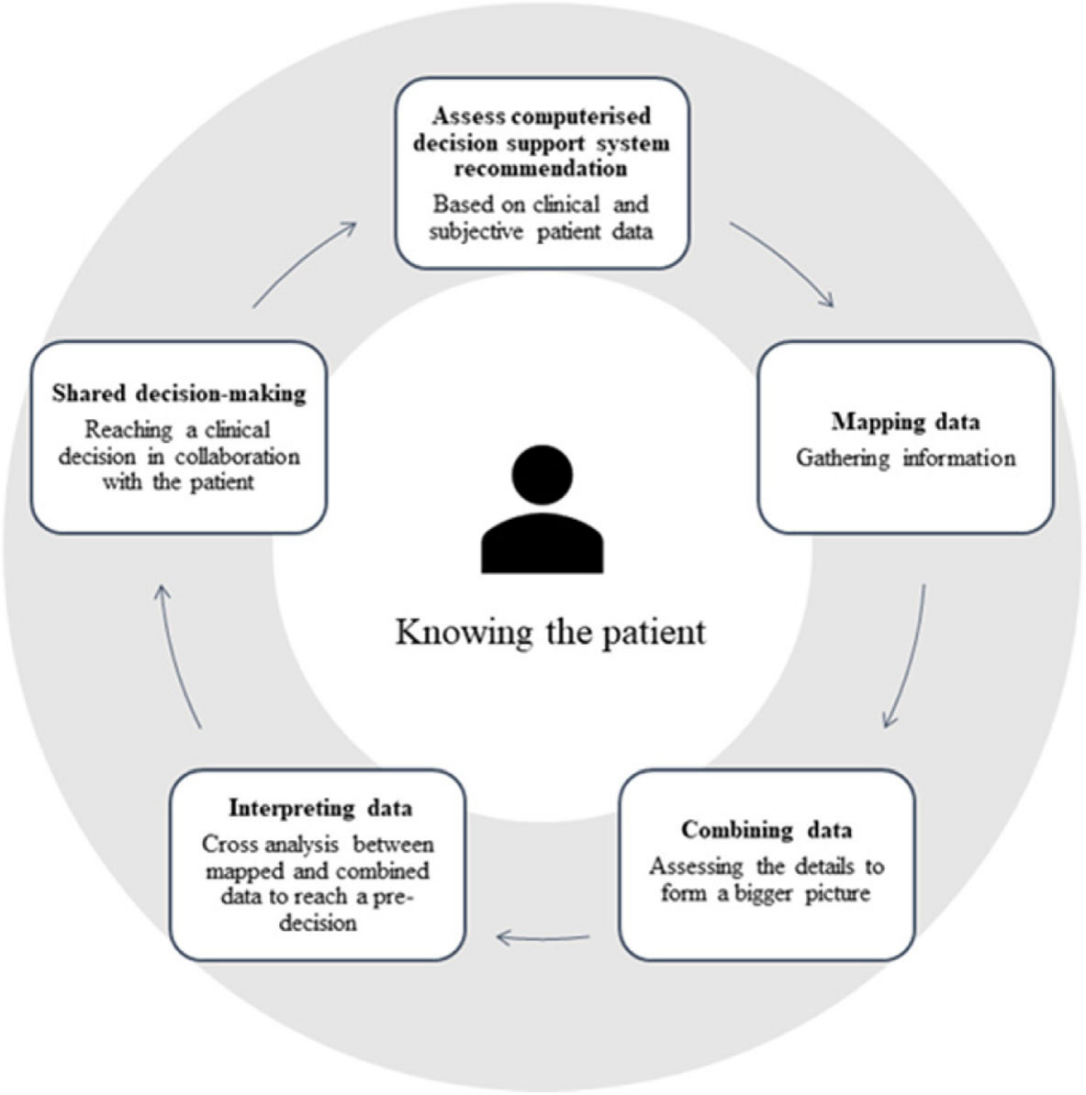
The five stages of telemedicine nurses’ circular reasoning and decision-making compared with that provided by the system alone

The process of TM nurses’ reasoning to assess health change was circular and dynamic and consisted of five stages: 1) assessing the CDSS recommendation, 2) mapping data, 3) combining data, 4) data interpretation to form a pre-decision, and 5) a final decision in collaboration with the patient (Table 5 and Fig. 1).

#### Assessing the CDSS recommendation

Nurses observed the CDSS recommendation and began the process of detecting symptom changes based on the patients’ normal health status. If the CDSS displayed a green colour-code, less time was spent assessing the patient. If the CDSS displayed a yellow or red colour-code, more time would be spent determining the type and extent of the health change. The nurses experienced that the CDSS often displayed inaccurate alerts. Therefore, they would frequently look beyond the colour-codes to assess the clinical data (saturation and heart rate), subjective symptoms (self-evaluated symptoms based on the questionnaire), and other patient information, regardless of the CDSS recommendations:

“I cannot look at that alone [the CDSS recommendation]. What colour is it [the system alert]? I need to look at what other diseases the patient has, his or her background, and how the patient has been recently [disease development].” (Nurse 1).

As part of the reasoning process, the nurses found recommendations from the software useful for initiating clinical reasoning (identifying health problems) and decision-making (prioritising follow-up needs). The system was used to clarify clinical information relating to each patient’s health to ascertain the meaning of any health changes. However, the CDSS did not adequately structure or provide sufficient clinical information regarding each patient’s health status. Consequently, system decisions were frequently overridden, and additional clinical information was sought.

#### Mapping, combining and interpreting data to reach a pre-decision

The data collected, including nursing documentation, which was based on medical records, medical histories, and the system recommendations, were diverse and multifaceted. Mapping of cues and combining clinical data contributed to a clinical overview, assisting interpretation of pattern changes specific to individual patient’s symptoms and disease development. These activities aided clarification of the patient’s current health status:

“…the picture, which was presented using the CDSS, was not clear enough for the nurses to evaluate, or to explicitly show the nurses the true picture of the patient’s health status. The nurses needed to search for more information by evaluating the available computerised information and analysing the data flow.” (Fieldwork observation).

The process of combining clinical data after mapping was complex and time-consuming involving assessing details to produce a complete overview or “bigger picture”:

“I need to combine the [system] information with something. I cannot look at the CDSS recommendation alone. I need to see the whole picture, the [patient’s] history, everything.” (Nurse 1).

Reflection characterised the combining data process, and a cross analysis of related clinical data between mapping, combining and interpreting. This cross analysis facilitated clarification of relevant information for each patient. During this process, the data gathered were combined and compared to eliminate bias in the system and to link the nurse’s in-depth knowledge of the patient with the clinical data. Using this process, the nurses could visualise an image of the patient and their current health status:

“The nurses formed a mental image of the patient and then gathered diverse information from earlier nursing documentation, and made mental notes. They then reviewed the system recommendation and detailed information to form the whole picture. The processes involved observing, analysing, reflecting, and recognising a pattern.” (Fieldwork observation).

The process of mapping, combining and interpreting data also helped to form possible future nursing scenarios including health developments, such as potential disease exacerbations. The nurses’ knowledge of each patient was constantly evolving, adding to the sum knowledge regarding the patients’ disease development. In addition, the patients’ experiences were considered highly-valued information. The more complex and unstable the patients’ health changes were, the more time was spent on clinical reasoning to reach a pre-decision.

#### From pre-decision to shared decision-making

Reasoning led to the formulation of a pre-decision regarding the patients’ health changes. If the pre-decision was based on deteriorating health, the nurse performed a video consultation to guide and advise the patient and produce a positive patient outcome. During each consultation, probing questions were asked to expand existing knowledge. Increasing the available knowledge led to clarification or adjustment of the pre-decision:

“If the CDSS indicated changes, or if the patient had indicated that their health had deteriorated, then the TM nurse needed to question the patient regarding the changes. For example, if the patient mentioned increased mucus, the nurse would ask by how much, what colour, and so on.” (Fieldwork observation).

Video consultation was important in aiding patient visualisation and was a significant additional component to the system recommendation when nurses were making decisions. Patient conversations became a part of the decision-making process, with the essential information provided increasing confidence in the clinical decision.

### The influence of the TM setting on nurses’ reasoning and decision-making processes

The TM environmental and technological aspects defined and structured the nurses’ work. Hence, the TM setting played an influential role in the nurses’ reasoning and decision-making processes.

#### Environment

Environmental aspects, such as the function and organisation of TM work, influenced the reasoning and decision-making processes. The nurses’ abilities to interact with the environment were based on their TM experiences. The nurse employed for the shortest time found the work more challenging:

“I must be honest. I am the one that has worked here for the shortest time, and I think that it has been difficult to get into this way of working.” (Nurse 3).

However, the TM intervention formed part of a newly developed project, and the recurring changes in the project development and management of new work tasks was a challenge for all the nurses:

“The project is evolving all the time. If you work on a ward, it is work [nursing] you have conducted for many years, and it is familiar and known, but here [at the TMC] it is a new way of working […] It is constantly changing. One live in such concentration because you experience things that are new. It is just how it is. It is not easy.” (Nurse 1).

Moreover, the nurses managed the TMC alone, which restricted collaboration between the nurses, physicians, and other healthcare professionals when making decisions:

“When I am alone at work I have to decide (concerning a patient) what I should or should not do… one feels very alone.” (Nurse 2).

Consequently, a lack of collaboration led to an absence of individual and shared goals for each patient. Therefore, the care plan and guidance provided to patients by the nurses was divergent. One nurse described how she was missing a more united and goal-setting collaboration:

“I would like us to share information, and collaborate more because we manage the TMC alone. There is no one else to talk to, except for the written reports […] And what it is we are supposed to achieve… the goal, what is the goal? A common goal, a goal for the individual patient, and intermediate goals...” (Nurse 3).

However, while the nurses found some of the aspects of TM work challenging, they also found it interesting, and the environment of working alone provided time and space to manage the patients regarding care planning, information gathering and consultations. In addition, time could be devoted to thoroughly conducting the reasoning process without distractions. Similarly, the TM setting enabled the development of strong nurse-patient relationships beyond the patient’s health, such as their personalities and life histories:

“You get a different relationship with patients [using TM and video conferencing] … somewhat closer, and it is much more personal.” (Nurse 2).

“A TM nurse gains insight into more than just the health status of each patient. One gets to learn so much more about the patient’s life, which is important for the clinical reasoning and decision-making processes in telemedicine work.” (Fieldwork observation).

#### Technology

The TM technology enabled the collection of daily clinical and subjective patient information, facilitated frequent distant monitoring of patients, and assisted in managing continuous follow-up. The TM nurses operated and managed three different screens representing different IT systems; one for assessing system recommendations (i.e. the CDSS), another for nursing documentation and electronic health record (EHR), and a third for performing real-time video consultations with the patients.

The patient information provided by the technology and being able to perform regular video-consultations supported the nurses’ reasoning and decision-making processes. However, at times the technology constrained the nurses’ work. The computer housing the CDSS and the EHR system were poorly integrated leading to increased time spent searching for relevant clinical information:

“The nurses are constantly handling two different software systems on two different computers. They spend much time searching for clinical information, leading to double entries. It seems ineffective and unnecessary, but such is the system.” (Fieldwork observation).

In addition, technical problems often interrupted the follow-up before addressing clinical problems. Consequently, the focus on medical urgency was displaced by the focus on technical difficulties. Fieldwork observations revealed that almost half of the daily conversations with the patients involved attempts to find a solution to technical problems. Once technical difficulties were resolved, patients were often tired and needed rest. Technical difficulties were mostly related to tablet malfunctions including the application or 3G/4G coverage, which wasted time and interrupted the nurses’ reasoning and decision-making processes:

“Technical failures occurred often. These were perceived as a burden on both the patient and nurse and the technical factor became the urgent problem. Technical problems overshadowed the patients’ health problems, and the reasoning and decision-making processes were disturbed. The TM nurses were tiring and sometimes postponed fixing the problem. It became a dilemma of time and resources.” (Fieldwork observation).

Consequently, video consultation follow-up was often replaced by telephone calls, meaning that the relevant visual information used for the reasoning and decision-making processes were difficult to implement. Furthermore, the recurring technical problems affected the nurses’ view towards their work and led to increasing frustration. The focus group interview revealed these frustrations with one nurse commenting:

“It is frustrating when you go all in. Telemedicine should be fast and simple, and almost all we do is sit fiddling with technical problems”. (Nurse 3).

Therefore, in some cases, the technology became a burden rather than support for reasoning and decision-making processes.

### Advancing beyond the system

The TM nurses’ reasoning and decision-making processes were influenced by the TM work and setting, which consisted of a highly technological environment, nurses over-riding the system recommendations, poorly integrated information systems, and clinical reasoning performed alone and at a distance from the patient. The TM setting, including the environmental and technological characteristics, both enabled and constrained nurses’ reasoning and decision-making processes. Although the setting, work and system provided challenges, it also provided time and support, influencing the nurses’ reasoning process. Daily clinical and subjective data, regular patient contact and continuous follow-up provided the nurses with detailed patient- and illness-specific knowledge cementing a close relationship with the patient. However, while the CDSS was intended to support the reasoning and decision-making processes, nurses regularly needed to advance beyond the system recommendations.

The TM nurses rarely used the recommendations from the CDSS in isolation, they combined and compared digital clinical and subjective patient data to identify any conflicts, then compared the outcomes using their long-term acquired patient knowledge. An acquired in-depth knowledge of each patient enabled the nurses to see past the system recommendation, termed “advancing beyond the system” (Fig. 1). Advancing beyond the system was a significant component of the reasoning process. Knowledge of the patient ensured competence in detecting CDSS biases and enabled the gathering of patient-specific information to detect individual changes in health status, producing a more accurate and nuanced decision. Figure 1 illustrates the circular and dynamic processes of reasoning and decision-making in the telemedicine setting (grey colouring shows settings). Knowing the patient is central, forming a foundation for reasoning at all five stages of the process.

## Discussion

The present study explored the process of TM nurses’ clinical reasoning when using a CDSS for the management of patients with COPD. The factors influencing the clinical reasoning and decision-making processes were also investigated. The study found that the TM nurses frequently advanced beyond the CDSS recommendations, and that the system both enabled and constrained the nurses’ reasoning process. Therefore, this discussion aims to provide a nuanced picture of the association between the reasoning and decision-making processes and various enablers and constraints of the TM setting.

In the present study, the CDSS enabled access to digital patient data and was helpful in initiating the reasoning process if the patient’s health status was unchanged. However, adverse changes in the patient’s health status produced inaccurate alerts, highlighting a limitation of the system. Consequently, reasoning and decision-making processes could not be based on the system recommendations alone. In addition, repeated technical errors and poorly integrated systems further constrained the nurses’ reasoning process as considerable time was spent gathering patient-related information. Therefore, the nurses would frequently override the system recommendation, which is also notable in previous studies [27, 33]. Furthermore, Oudshoorn [10] found that TM nurses often developed workarounds to provide care according to their standards and acquired patient knowledge. Cappelletti et al. [26] suggests that the methods of information gathering and sharing can influence the type and depth of the reasoning process. Consequently, it is important that the CDSS facilitates efficient access to information [53], and provide accurate recommendations [23], especially for remote care.

The present study revealed that in the remote care, the TM nurses were dependent of patient information made available by technology. Reasoning in a TM setting implies a different approach to patients than in traditional nursing care, entailing advanced care performed remotely and supported by information and communication technology [10, 11]. Studies have shown that systems generating alerts are effective for improving clinical practice [19], patient care [19, 31] and chronic disease management (28,29). By contrast, Miller et al. [22] suggests that CDSSs is an emerging technology, with poor system uptake and use. Therefore, and to further support nurses’ reasoning using CDSS, system development should be both theoretical [54] and evidence-based [39, 55], including knowledge reflecting nursing expertise [56].

The TM nurses in the present study were alone in making decisions concerning patients, and, although frequently overriding the CDSS, were simultaneously dependent on the information the CDSS provided. Several studies show that absence of the patient and of visual cues in the TM setting reduces the reliable information sources [10, 32–34]. The present study shows that video-mediated contact is a key in enhancing the reasoning and decision-making processes facilitating individualised follow-up, patient involvement, and forming close nurse-patient relationships. Regular patient contact provided an opportunity to ask patients probing questions and to gather extensive clinical and subjective information, beyond what provided by the CDSS. This indicates that performing regular video consultations in a TM setting provides additional and valuable patient information and strengthens the nurse-patient relationship. By contrast, technical problems, resulting in telephone-mediated consultations constrained the reasoning process in the TM setting.

Personal patient knowledge was an essential factor in understanding a patient’s healthcare needs in a remote care setting. Knowing the patient has been proven to influence the reasoning process [24–27]. An adequate knowledge of the patients, as well as knowledge of their disease, can provide valuable information on pattern recognition and responses [10, 25, 26]. In line with these studies, the present study demonstrated that in-depth knowledge of each patient provided the nurses with the ability to “advance beyond the system” and detect health changes beyond the recommendations provided by the CDSS. These findings are supported by Edwards [24], who showed that knowing the patient provides the nurse with a standard set of clinical data, enabling the variation elimination and providing more accurate and controlled information.

The reasoning process in the present study promoted patient involvement through shared decision-making. Nurse-patient conversations were necessary to verify and elaborate on the initial reasoning process. Patients’ experiences of illness and symptoms could help to verify or adjust the pre-decision, indicating that the patients’ illness narratives and subjective symptoms often played an equally significant role as the clinical measurements displayed by the CDSS. Patient involvement and shared decision-making were also reported in studies that focused on the patients’ experience when receiving TM [9, 57]. However, several aspects can influence the patients’ preference for shared decision-making, such as the experience of and involvement with illness and the nurse-patient relationship [58]. Consequently, nursing practice and research need to account for the patients’ participation in the decision-making process [59] when using CDSS.

The present study found that the continuous flow of digital data provided by the CDSS, might facilitate a detailed understanding of the individual COPD patient’s health and provide a better overview of chronic disease development unique to an individual. However; long-term management of patients with COPD often involve different illness progression rates, comorbidities, and patient anxieties. Also, patients’ symptoms for chronic illness are often subtle [10] and the physical and mental limitations of patients with COPD are often unpredictable [9, 14], leading to diverse [60, 61] and complex decision-making [21]. Gerdes et al. [48] found limitations in the long-term monitoring for patients with COPD when using CDSS in relation to day-to-day interpretation of the patients’ health status. This indicates that nurses are essential for understanding and interpreting the complex, subtle and unpredictable health changes in long-term management of COPD. TM monitoring transforms care into a continuous process [10, 62], which facilitates the notion of a continuum of care [63], supporting the role of TM nurses in the reasoning and decision-making processes when using CDSS.

### Strengths and limitations

The present study has limitations. First, the sample size of three nurses was small. The ideal would have been to include more participants as well as to perform extended observations. However, even though the nurses did not want to participate further, a rich data material was collected. Savage [43] indicates that ethnography could be used as a method for every scale, small or large, where social research is performed in a natural, everyday setting.

Secondly, fieldwork might affect the participants’ behaviours and actions [42], and the researchers objectivity to the field can both affect the researcher, data collection and interpretation [64]. During the observations, the first author used a reflective approach, acknowledging bias, preferences, and preconceptions. Further, the first author is a registered nurse, and many of the skills that a nurse has acquired is similar to what an ethnographer holds, for example features such as listening, interviewing, observing, reflecting and interpreting on multiple levels at the same time, and with a conscious use of oneself. This illustrates that a nurse with professional knowledge and experience can offer security in the TM setting, thus experiencing successful interaction [65]. In addition, with the use of the think-aloud technique, the author was positioned next to, rather than facing, the participants to minimise influence on the participants.

The present study used a combination of different data collection methods to strengthen the study as it provides a more nuanced and complete picture of the phenomenon investigated [64]. Also, the focus group interview promoted a meeting point and discussion between the nurses, which facilitated a broader experience regarding the TM context, and of the use of the CDSS. The findings of a qualitative study are difficult to generalise, however the findings can be transferred to similar settings [51].

## Conclusions

In the TM setting, nurses’ reasoning supported by a CDSS was enabled by the continuous flow of computerised clinical data, regular video-mediated contact with the patients and shared decision-making, all of which strengthened in-depth knowledge of the patients, acting as a foundation for nurses’ reasoning and decision-making processes. Regular patient contact via TM promoted a continuum of care, supporting the role of TM nurses’ in the long-term management of COPD. Nevertheless, nurses frequently advanced beyond the system recommendations, which indicates that future research is needed to develop more accurate algorithms, increase system maturity and improve the integration of digital clinical information with clinical experiences. Future TM services should be organised in a way that maintains the continuous flow of clinical data, involve regular video-mediated contact and promote shared decision-making to support nurses’ reasoning.

## Abbreviations

CDSS: Computerised decision support system
COPD: Chronic obstructive pulmonary disease
EHR: Electronic health record
TM nurses: Telemedicine nurses
TM: Telemedicine
TMC: Telemedicine centre
U4H: United4Health
